# Melatonin Increases the Sensitivity of Hepatocellular Carcinoma to Sorafenib through the PERK-ATF4-Beclin1 Pathway

**DOI:** 10.7150/ijbs.32550

**Published:** 2019-07-21

**Authors:** Bei Zhou, Qianqian Lu, Jiatao Liu, Lulu Fan, Yu Wang, Wei Wei, Hua Wang, Guoping Sun

**Affiliations:** 1 Department of Oncology, the First Affiliated Hospital of Anhui Medical University, Hefei 230022, Anhui, China;; 2 Department of Pharmacy, the First Affiliated Hospital of Anhui Medical University, Hefei 230022, Anhui, China;; 3 Institute of Clinical Pharmacology, Anhui Medical University, Hefei 230032, Anhui, China;; 4 Institute for Liver Diseases of Anhui Medical University, Hefei 230032, Anhui, China.

**Keywords:** Hepatocellular carcinoma, Sorafenib, Melatonin, Endoplasmic reticulum stress, Autophagy, Apoptosis resistance

## Abstract

The mechanisms of resistance to the targeted drug sorafenib in the treatment of hepatocellular carcinoma (HCC) are poorly understood. The purpose of this study was to investigate the mechanism of sorafenib resistance and to elucidate the role of melatonin in overcoming sorafenib resistance. We first observed that sorafenib induced endoplasmic reticulum (ER) stress and activated autophagy in HCC, and the inhibition of ER stress and autophagy by specific inhibitors (PBA, TUDC and 3-MA) increased sorafenib-induced apoptosis, indicating that cells resist apoptosis by inducing ER stress and autophagy in the presence of sorafenib. Furthermore, specimens from patients with HCC revealed a close relationship between ER stress and autophagy, as demonstrated by the high correlation between expression of the autophagy-associated protein Beclin1 and expression of unfolded protein response (UPR) pathway proteins, especially PKR-like ER stress kinase (PERK); moreover, patients with combined expression of PERK and Beclin1 had more advanced disease (higher clinical stage) and a shorter overall survival time. ER stress inhibitors significantly blocked sorafenib-induced autophagy, selective knockdown of PERK and activating transcription factor 4 (ATF4) expression reduced sorafenib-induced autophagy activity compared with knockdown of the other two UPR pathways, and silencing ATF4 inhibited the expression of Beclin1. These results suggest that autophagy is downstream of ER stress and that the PERK-ATF4-Beclin1 pathway plays a role in ER stress-related autophagy. Interestingly, a low concentration of melatonin increased the sensitivity of HCC to sorafenib by inhibiting autophagy through the PERK-ATF4-Beclin1 pathway. Taken together, our findings suggest that cotreatment with sorafenib and melatonin is a potential therapy for HCC. Furthermore, ER stress-related autophagy plays key roles in apoptosis resistance. Therefore, targeting the PERK-ATF4-Beclin1 pathway may prove instrumental in HCC therapy.

## Introduction

The rate of death from hepatocellular carcinoma (HCC) is increasing faster than the rate of death from any other cancer, likely due to the lack of effective treatment and high drug resistance 1, 2. Although treatment options are limited, the multitarget tyrosine kinase inhibitor (TKI) sorafenib has been used to treat HCC, thyroid cancer, and renal cell carcinoma, resulting in an increased median survival of HCC patients from 7.9 to 10.7 months 3-5. Although initial treatment can achieve an unexpected response, sorafenib-treated liver cancer lumps rarely disappear completely, and the positive effects are usually transient; HCC patients show radiological progression after 4-5 months of sorafenib treatment and ultimately experience sorafenib resistance 6, 7. Therefore, elucidation of the cause of sorafenib resistance in the treatment of HCC and improvement of its therapeutic effect are currently hot topics.

Studies have demonstrated that endoplasmic reticulum (ER) stress and autophagy mediate chemoresistance in tumor cells 8, 9. ER stress is the pathological accumulation of unassembled and misassembled peptide chains in the lumen of the ER resulting from stimulation by various factors such as ischemia, hypoxia, and drugs that impair ER function 10. Under ER stress, the molecular chaperone GRP78 is upregulated, depolymerizes and activates three sensory proteins on the ER membrane, including PERK, activating transcription factor 6 (ATF6) and inositol-requiring transmembrane kinase and endonuclease 1 (IRE1), which activate the corresponding downstream pathway to modulate the expression of relevant target genes that can reduce the load of proteins requiring processing and folding in the ER lumen and increase the capacity for protein folding, processing, and ER-associated degradation (ERAD). This response that maintains the stability of the intracellular environment is called the unfolded protein response (UPR) 11, 12. Modest ER stress promotes an adaptive mechanism that mediates HCC resistance to apoptosis through various signaling pathways. However, excessive and sustained ER stress can directly lead to ER stress-related apoptosis 13. Autophagy is a highly evolutionarily conserved catabolic process that is widely present in eukaryotic cells and plays a critical role in various stress conditions, including starvation, hypoxia, drug treatment, and oxidative stress 14, 15. Autophagy is an important mechanism of metabolic degradation at the cellular level that controls the survival of cells under stress; in this process, redundant organelles and proteins are isolated by double-membrane autophagosomes that fuse with lysosomes, in which the contents are digested to recycle monosaccharides and amino acids 16, 17.

Studies have indicated that ER stress can compensate for autophagic activity in cells, and activated autophagy eliminates dysregulated proteins induced by ER stress, suggesting that ER stress may be an initiation factor for autophagy activation 18, 19. The connection between ER stress and autophagy induced by sorafenib in HCC cells is currently unknown. It is necessary to verify the role of ER stress and autophagy in the resistance of HCC cells to apoptosis and to investigate the molecular mechanisms relating autophagy and ER stress in the context of sorafenib treatment.

Melatonin, a physiological hormone that is primarily secreted by the pineal gland, has a wide range of biological functions, including physiological regulation, antioxidation activity, anti-inflammatory activity, immune regulation, and antitumor activity 20, 21. Numerous studies have indicated that melatonin possesses chemotherapeutic potential as an adjuvant in multiple cancers and can regulate signaling pathways governing cell proliferation, apoptosis, survival, ER stress and autophagy 22-24. Several studies have proposed possible mechanisms by which melatonin increases the efficacy of chemotherapy drugs and overcomes chemotherapeutic resistance in cancer 23, 25. These reports are consistent with our previous study showing that melatonin can overcome apoptosis resistance and increase the sensitivity of HCC to doxorubicin 26. However, studies on the synergistic effects of melatonin with molecular-targeted drugs and their mechanisms are limited. In this research, we provide experimental evidence that melatonin enhances the sensitivity of liver cancer cells to sorafenib by inhibiting autophagy induced by ER stress.

## Methods

### Reagents and antibodies

Primary antibodies applied in this study including antibodies against PERK, Bax, ATF6, Bcl-2, CHOP (Bioworld Technology, BS2156, BS2538, BS1034R, BS1511, BS1136), IRE1α, XBP1s (Cell Signaling Technology, 14C10, D2C1F), GRP78 (Arigo Biolaboratories, ARG54027), ATF4 (Abnova GmbH, 2B3), LC3B (Sigma, L7543), P62, Beclin1 (Abcam, ab179800, ab32503). Reagents including tunicamycin, 3-methyladenine, melatonin (Sigma Chemical, T7765, M9281, M5250), PBA, TUDC (Selleckchem, S4125, S7896), TUNEL system (Roche), the Annexin V-FITC/PI Apoptosis Detection Kit (BD Biosciences).

### Cell culture

The HepG2, 7721, Huh7 and LO2 cell lines were purchased from the Shanghai Cell Bank (Chinese Academy of Sciences, Shanghai, China). The basic cell culture medium was high-glucose DMEM supplemented with 10% fetal bovine serum (FBS) and 1% penicillin-streptomycin (PS). The cells were cultured in an incubator with 5% CO_2_ at 37℃.

### Human hepatocellular carcinoma specimens

Seventy-two HCC cases were derived from patients who underwent radical resection of liver cancer at the Affiliated First Hospital of Anhui Medical University from 2001 to 2017. Patients who received chemotherapy or other treatments before surgery were not included in the study. Clinical parameters included hepatitis history, tumor size, overall survival, clinical stage, and degree of tumor differentiation. All clinicopathological information was collected by reviewing electronic medical records and pathology reports. All clinical specimens were obtained from patients with written informed consent. The Ethics Committee of Anhui Medical University (Anhui, China) authorized the study and acquisition of clinical specimens.

### Tissue microarray construction

HCC specimens fixed with formalin and embedded in paraffin were collected from the Department of Pathology, the First Affiliated Hospital of Anhui Medical University. The target regions of the cancer and adjacent normal tissues were selected for microarray construction according to the HE staining results. Representative cores (1 mm) were excavated from the target area, and the acceptor paraffin block was secondarily embedded and made into a tissue microarray.

### Immunohistochemical analysis

The tissue chip was deparaffinized and incubated in 3% hydrogen peroxide to block intrinsic peroxidase activity, and the sections were heated in 0.01 mol/L sodium citrate buffer for antigen retrieval. Next, the tissue chip was incubated with the specific primary antibody for 1 h at room temperature and then with the corresponding secondary antibody and peroxidase-conjugated streptavidin. Subsequently, the slides were stained with DAPI and hematoxylin. The staining intensity was graded under a microscope, and the ratio of positively stained cells to the total number of tumor cells in the field of view was calculated. A total score greater than 2 was considered positive.

### Western blot analysis

Briefly, the cells were lysed on ice for 30 min. Lysates were centrifuged at low temperature and high speed (14000 rpm) for 10 min. The protein content of the supernatant was detected by the BCA assay. Purified protein samples were incubated with 4× loading buffer and heated at 95°C for 10 min. Protein samples from each group were separated by SDS-PAGE and transferred onto PVDF membranes under constant current (Millipore, Bedford, MA, USA). The protein-loaded membranes were immersed in 5% nonfat milk to block nonspecific proteins and then incubated with specific primary antibodies at 4°C overnight, followed by incubation with the corresponding secondary antibody (1:50,000 dilution) at 37°C for 2 h. A hypersensitive luminescent reagent (ThermoFisher, USA) was used to visualize the protein bands, and the signals were captured with an Image Quant™ LAS-4000 Mini Developer (Fuji, Japan). The grayscale value of each immunoreactive band was determined using ImageJ software (US National Institutes of Health) for quantitative analysis.

### Flow cytometry

Flow cytometry was performed using two channels to detect annexin V-FITC and PI in order to determine the proportion of apoptotic cells. Drug-treated suspension and adherent cells were collected into a flow tube and then incubated with 5 μl of annexin V-FITC and 5 μl of PI in a dark room for 15 min at room temperature. The data were acquired using a Beckman CytoFLEX LX flow cytometry system (Beckman Coulter, USA) and were processed using CytExpert software (Beckman Coulter, USA).

### Transmission electron microscopy

Transmission electron microscopy analysis of autophagosome formation is the classic method to study autophagy. Briefly, HepG2 cells were plated in 100-mm-diameter dishes. After treatment with the indicated compound for the indicated time, the cells were collected and centrifuged at 3,000 rpm. Thereafter, the cells were fixed in 2.5% glutaraldehyde (SPI Supplies) and 1% osmium tetroxide. After the cell pellet was dehydrated in an alcohol gradient, it was embedded in epoxy resin and cut into ultrathin sections (70 nm) using a NOVA ultramicrotome (LKB Biotechnology), followed by double staining with Reynolds' lead citrate and 1% uranyl acetate. Finally, data were acquired by transmission electron microscopy (JEM-1230; Jeol Ltd, Japan).

### Immunofluorescence

HepG2 cells were uniformly seeded on sterile coverslips in 24-well culture plates for adherent growth. After the indicated treatment for a specified time, the cells were sequentially fixed in 4% paraformaldehyde and permeabilized with 0.5% Triton X-100, and then, the coverslips were immersed in 5% BSA at room temperature for 1 h. Subsequently, the pretreated samples were incubated with primary antibodies (dilution 1:100) overnight at 4°C and then with goat anti-mouse/rabbit secondary antibody (dilution 1:100) in the dark for 2 h. Finally, following incubation with DAPI for nuclear staining, the cells were imaged using a confocal microscope.

### Detection and quantification of acidic vesicle organelles (AVOs) by acridine orange staining

After the cells were treated as indicated, acridine orange dye was added to each well at a final concentration of 1 μg/ml, and the cells were incubated for another 15 min at 37°C in the dark. Photographs were captured using an inverted fluorescence microscope (Olympus, Tokyo, Japan). The orange fluorescence intensity was detected by flow cytometry to estimate the number of cellular acidic vesicles. Cells stained with acridine orange were trypsinized and then collected in phenol red-free growth medium. The fluorescence emission (λ_ex_: 488 nm; λ_em_: 670 nm) of the sample was detected using a CytoFLEX flow cytometer (Beckman, USA). The data were analyzed using CytExpert (Beckman, USA).

### Small interfering RNA (siRNA) transfection

HCC cells were plated and grown in 6-well plates before transfection at approximately 50% confluence. The cells were transfected with 50 nmol/L siRNA targeting IRE1α, PERK, ATF6, or ATF4 or control nonspecific siRNA using Lipofectamine® 2000 in accordance with the manufacturer's protocol. After 48 h of incubation, the transfected cells were used for subsequent experiments.

### TUNEL assay

The cells were adjusted to the appropriate density, uniformly seeded on coverslips in a 6-well plate, fixed with 4% paraformaldehyde, and incubated with 3% hydrogen peroxide to block intrinsic peroxidase activity. Next, the cells were permeabilized on ice for 10 min with 0.1% Triton X-100 (soluble in 0.1% sodium citrate). The specific steps of TUNEL staining were carried out in accordance with the product instructions. Images were captured using a Nikon ECLIPSE 80i biology microscope.

### MTT assay

After trypsinization, the cells were plated in a 96-well plate at 10,000 cells/well. After a certain period of drug treatment, 20 μl of MTT was added to each sample, and the plate was placed in the incubator for another 4 h. Next, the supernatant was discarded, 150 μl of DMSO was added to each well, and the plate was shaken for 10 min in the dark to sufficiently dissolve the formazan crystals. The absorbance was measured at 490 nm using a multifunctional microplate reader (Bio-Tek Instruments Inc., Winooski, VT, USA).

### Statistical analysis

SPSS 16.0 (SPSS Inc., Chicago, IL) was used to performed the statistical analysis. Each result was acquired from more than 3 independent experiments, and data are presented as the mean ± standard deviation. A two-tailed t-test was used to analyze significant differences between 2 independent groups. One-way analysis of variance was used for multiple comparisons to analyze significant differences among > 2 groups. P < 0.05 was recognized as statistically significant.

## Results

### A low concentration of melatonin increases the sensitivity of HCC cells to sorafenib

**Figure 1:**
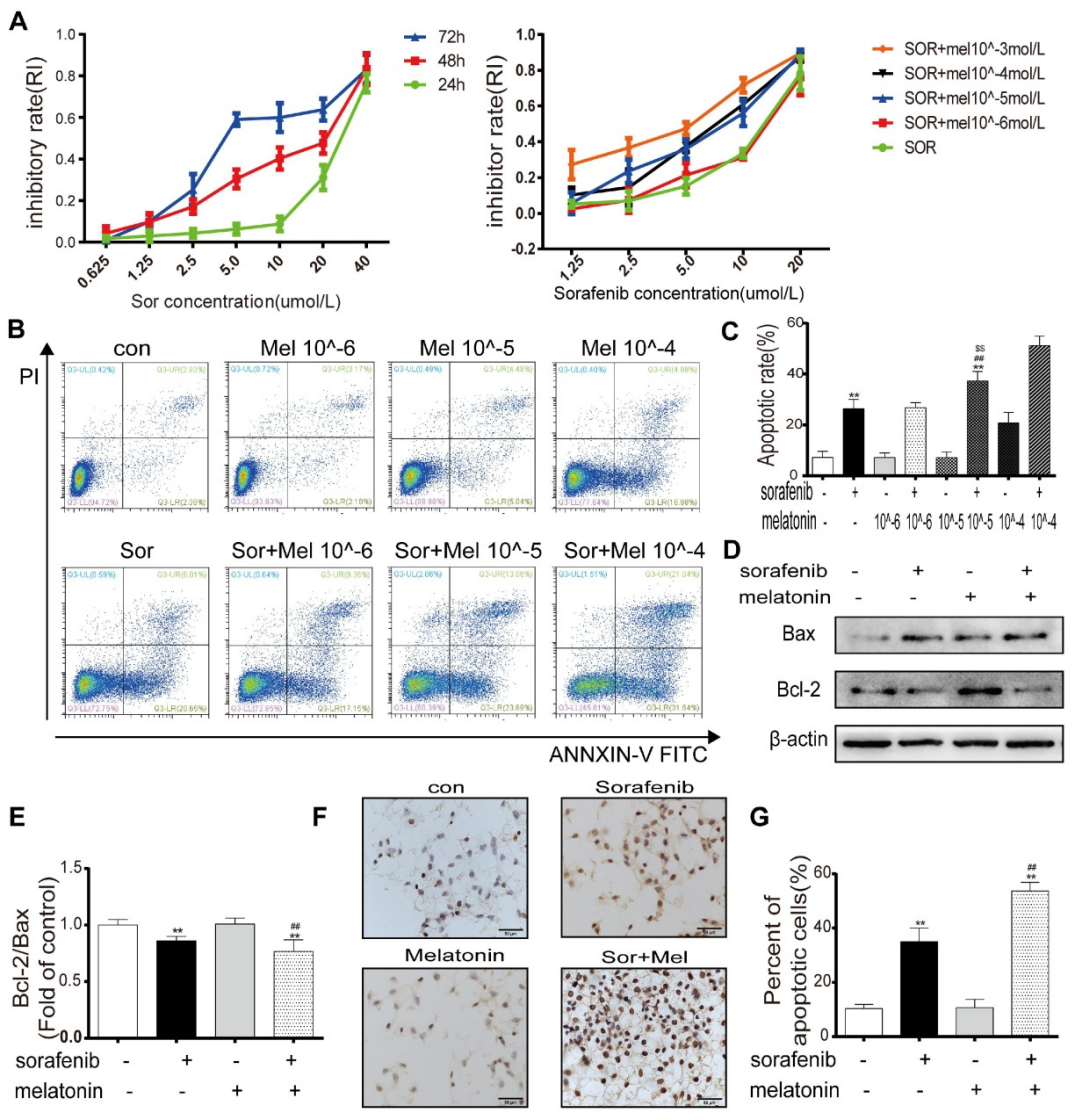
** Low concentration of melatonin increases the sensitivity of hepatocellular carcinoma cells to sorafenib** (A) Cells were exposed to sorafenib (0.625, 1.25, 2.5, 5.0, 10, 20, 40μM) for 24, 48, 72h (Left). Cells were exposed to sorafenib (1.25, 2.5, 5.0, 10, 20μM) with melatonin (10^-6^, 10^-5^, 10^-4^, 10^-3^mol/L) for 48h (Right). Cell viability was examined by the MTT assay. (B,C) Flow cytometry analysis of PI and AnnexinV in HepG2 cells cotreatment sorafenib (10μM) with melatonin (10^-6^, 10^-5^, 10^-4^mol/L) for 48h. (D,E) Expression levels of BCL2, BAX in HepG2 cells cotreatment sorafenib with melatonin. (F,G) TUNEL staining analysis detect cell morphology of apoptotic cells. **P < 0.01 compared to Control, ^##^P < 0.01 compared to Sorafenib. ^$$^P < 0.01 compared to Sorafenib +Melatonin 10^-6^mol/L(n = 3).

To determine whether sorafenib affects cell viability, HepG2 cells were treated with different concentrations of sorafenib for 24, 48, and 72 h. The MTT assay results demonstrated that the half-maximal inhibitory concentrations at these times points were 26.847 μM, 12.156 μM, and 7.217 μM (Figure 1A). To evaluate the potency of melatonin as a sorafenib sensitizer, the synergistic effect of melatonin and sorafenib was evaluated in HepG2 cells. Cell viability was determined by the MTT assay after treatment with sorafenib with or without melatonin at various concentrations for 48 h (Figure 1A). The MTT results indicated that exposure to sorafenib together with melatonin at 10^-5^~10^-3^ mol/L generated significant HepG2 cell death. It is worth noting that a low concentration of melatonin (10^-5^ mol/L) significantly increased HepG2 cell death at sorafenib concentrations of 5 μM and 10 μM. Consistent with the MTT assay results, the annexin V-FITC/PI apoptosis results showed that 10^-5^ mol/L melatonin did not increase the apoptosis of HepG2 and Huh7 cells as a single agent but significantly enhanced sorafenib-induced apoptosis (Figures 1B-1C, Figures S1A-1B). Therefore, 10^-5^ mol/L melatonin was used to analyze synergism with sorafenib in subsequent experiments. Similarly, Western blot results revealed that the ratio of bcl-2/bax was significantly downregulated in HepG2 cells exposed to both sorafenib and melatonin compared with sorafenib alone (Figures 1D-1E). Furthermore, TUNEL staining showed that cotreatment with sorafenib and melatonin caused a noticeable increase in the ratio of apoptotic cells, supporting the flow cytometry results (Figures 1F-1G). Overall, these results indicate the ability of melatonin to sensitize cells to sorafenib-induced apoptosis.

### Sorafenib activates ER stress and autophagy in HCC

**Figure 2:**
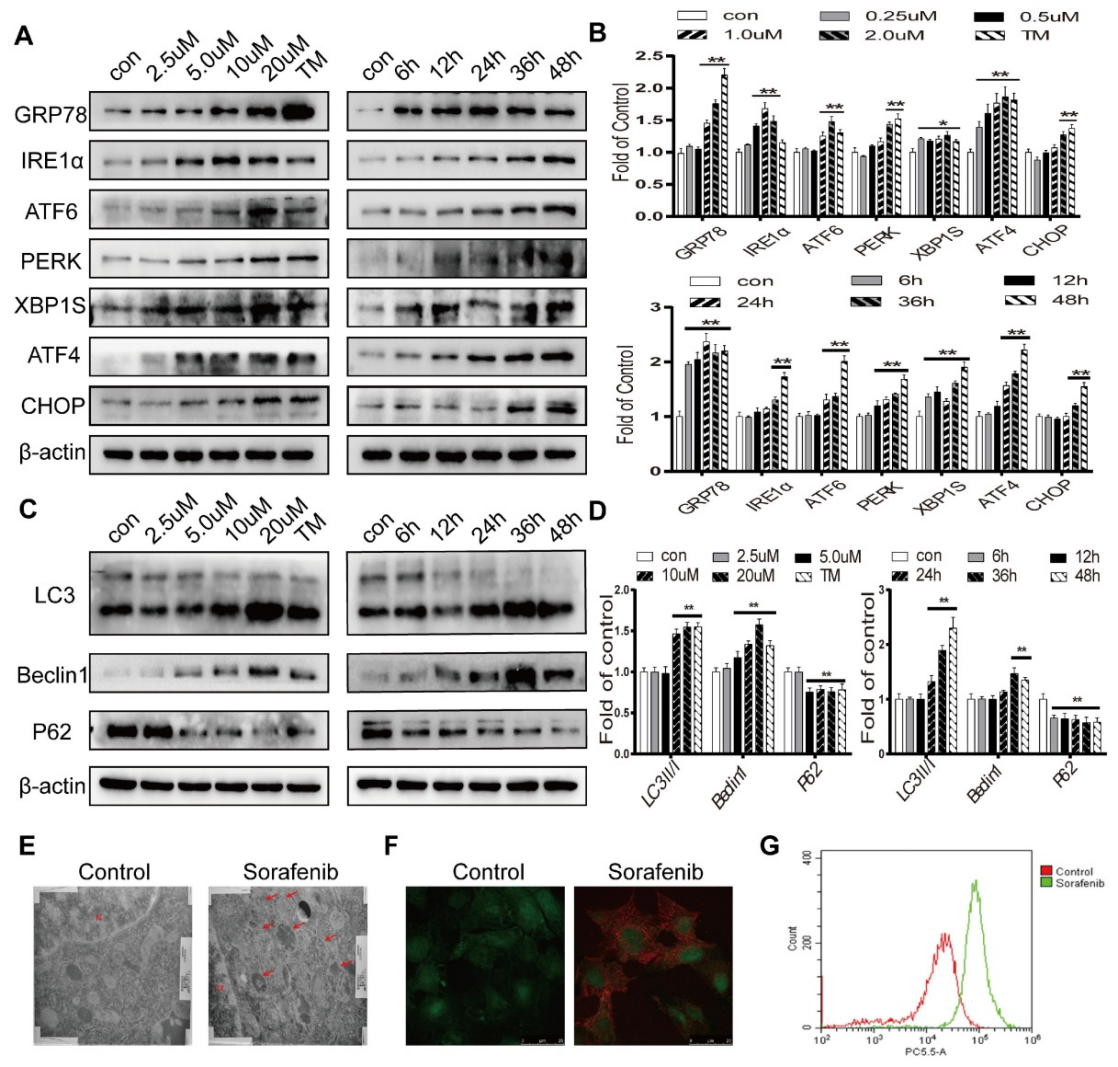
** Sorafenib activates ER stress and autophagy in HCC.** (A, B) The expression levels of the ER stress markers GRP78, IRE1α, ATF6, PERK, XBP1S, ATF4 and CHOP were analyzed by Western blot. (C, D) The expression levels of the autophagy markers LC3, Beclin1 and P62 were analyzed by Western blot. (E) Observation of autophagosomes with double-membrane structures under electron transmission microscopy (arrows). (F) Acridine orange images were obtained 48 h after sorafenib treatment (10 μM). (G) AVO quantification by flow cytometry analysis. *P < 0.05, **P < 0.01 compared to control. (n = 3).

To determine whether sorafenib induces ER stress and autophagy, Western blot analysis was first conducted. Tunicamycin, a classic ER stress inducer, stimulated an increase in the expression levels of GRP78, CHOP and the UPR target genes IRE1α, ATF6, PERK, ATF4, and XBP1S. Sorafenib also significantly increased the protein expression levels of these ER stress markers (Figures 2A-2B). The conversion of the free form LC3 (LC3-I) into the constitutive and autophagosome-related form (LC3-II) is a significant indicator of autophagy activation. Our results showed that LC3-II was increased continuously in HepG2 cells exposed to a concentration curve of sorafenib for various times. The autophagy-related protein Beclin1, which regulates autophagy, is associated with the production of phosphatidylinositol 3-phosphate (PtdIns(3)P) which related to the consecutive colocalization of other autophagy-associated proteins that coordinate autophagosome formation 27 was obviously increased, and P62 was clearly decreased after treatment with sorafenib (Figures 2C-2D). Consistently, electron microscopy revealed that multimembrane vacuolar structures, which are considered microscopic characteristics of autophagosomes, were increased in sorafenib-treated cells (Figure 2E). Sorafenib also stimulated the accumulation of AVOs in HepG2 cells, as evidenced by acridine orange staining (Figure 2F). AVO quantification by flow cytometry analysis indicated that sorafenib increased the autophagic vesicle content proportional to the red fluorescence intensity over the 48-h period subsequent to drug exposure (Figure 2G).

### Inhibition of ER stress and autophagy can increase sorafenib-induced apoptosis

**Figure 3:**
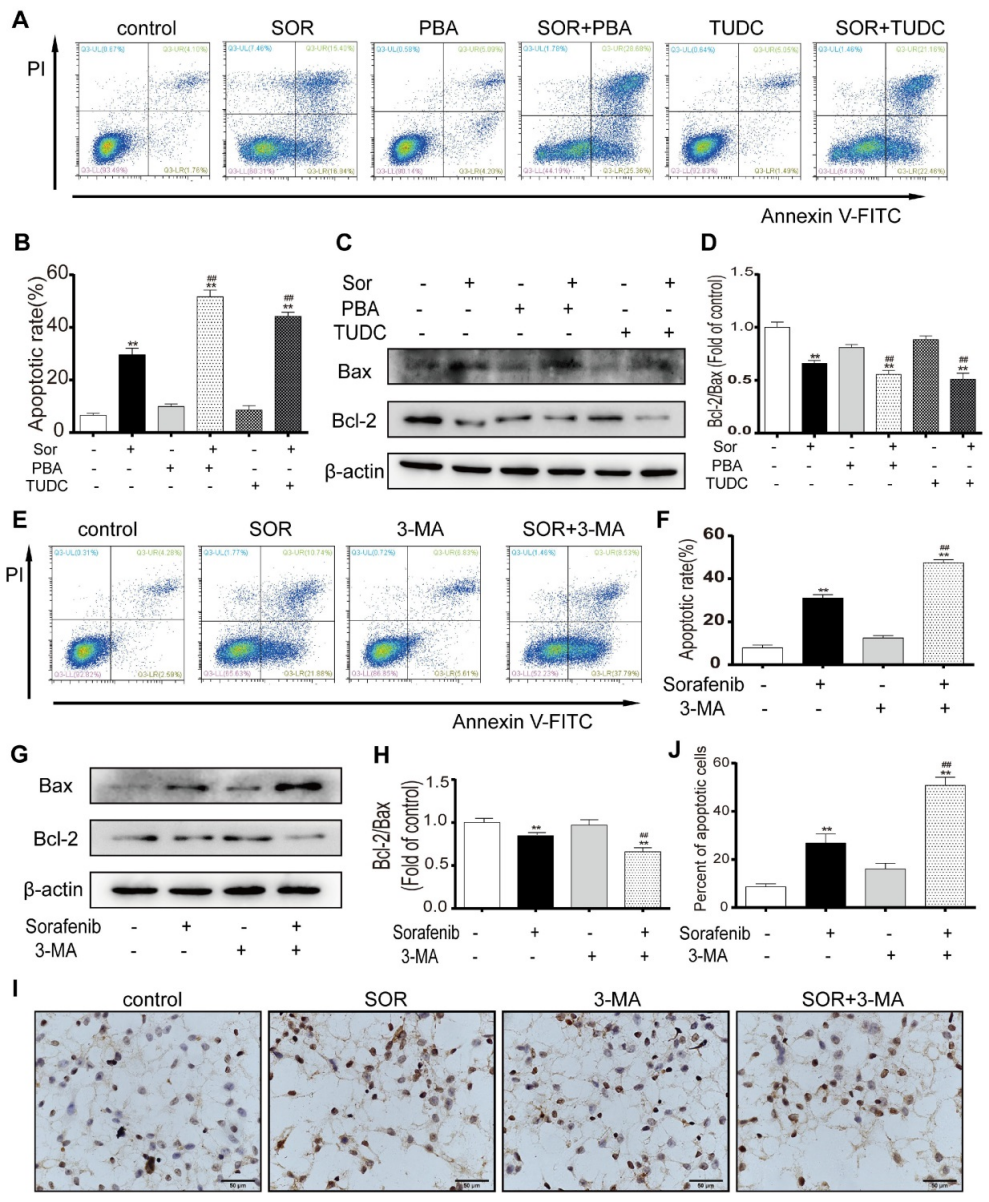
** Inhibition of ER stress and autophagy can increase sorafenib-induced apoptosis.** (A, B, E, F) Flow cytometry analysis of the percentage of apoptotic HepG2 cells cotreated with sorafenib (10 μM) and PBA (1 mM) or TUDC (1 mM) (A, B) or 3-MA (1 mM) (E, F). (C, D, G, H) Expression levels of BCL2 and BAX in HepG2 cells cotreated with sorafenib and PBA or TUDC (C, D) or 3-MA (G, H). (I, J) TUNEL staining analysis of the morphology of apoptotic cells. **P < 0.01 compared to control; ^##^P < 0.01 compared to sorafenib. (n = 3).

To assess the effect of sorafenib-induced ER stress activation on apoptosis, an ER stress inhibitor was used to observe the change in the apoptotic rate of HCC cells. PBA and TUDC act as molecular chaperones to bind unfolded proteins to inhibit ER stress and attenuate the UPR. Flow cytometry assays for PI and annexin V indicated that sorafenib combined with PBA or TUDC significantly increased HepG2 cell apoptosis (Figures 3A-3B). Furthermore, the expression of the apoptosis marker BCL2 significantly declined in response to cotreatment with sorafenib and PBA or TUDC compared with treatment with sorafenib alone. In contrast, the expression levels of the proapoptotic marker BAX were significantly increased (Figures 3C-3D). To examine whether autophagy has protective effects on HCC cells by resisting apoptosis, annexin V-FITC/PI apoptosis assays were performed in HepG2 cells using flow cytometry. The scatter plot results showed that 3-MA significantly increased sorafenib-induced apoptosis (Figures 3E-3F). Consistently, our Western blot results showed that the bcl-2/bax ratio was clearly downregulated by the combination of sorafenib and 3-MA compared with sorafenib alone (Figures 3G-3H). Furthermore, TUNEL staining was performed using HepG2 cells. Consistent with the flow cytometry assay results, the number of TUNEL-positive cells, which indicates apoptosis, was visibly increased after the addition of 3-MA (Figures 3I-3J). These findings indicate that the addition of the ER stress inhibitor and autophagy inhibitor could promote sorafenib-induced apoptosis.

### Characterization of HCC specimens

**Figure 4:**
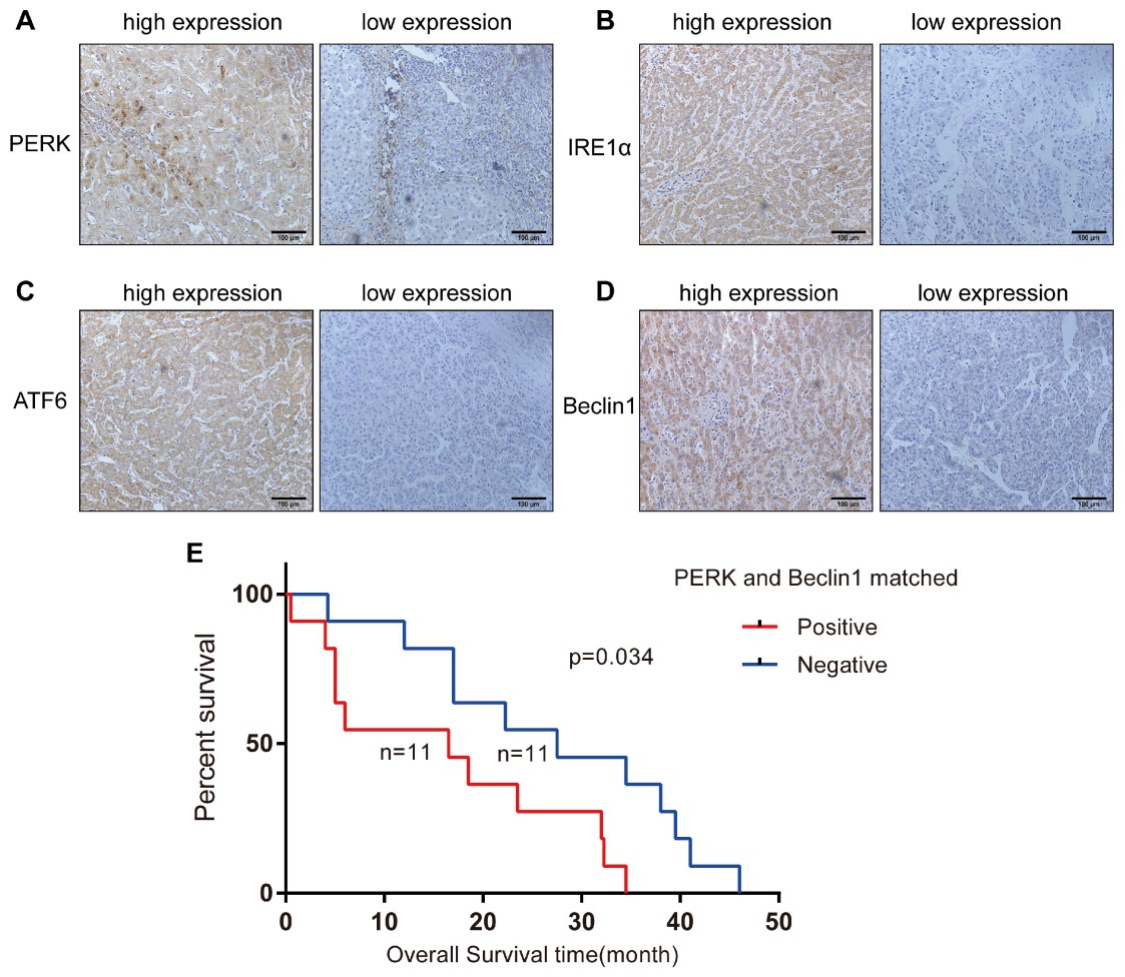
** ER stress and autophagy are related in HCC specimens, and patients with both PERK and Beclin1 expression have a shorter overall survival time.** (A-D) Expression of PERK, IRE1, ATF6, and Beclin1 in HCC specimens (streptavidin-peroxidase ×200). (E) Comparison of Kaplan-Meier cancer-specific survival curves between the matched PERK- and Beclin1-positive and PERK- and Beclin1-negative groups.

To investigate whether ER stress and autophagy are related in HCC, we analyzed 72 HCC specimens by immunohistochemical staining to evaluate the expression levels of IRE-1α, ATF-6, PERK and Beclin1 in the cytoplasm or nucleus. We noticed that Beclin1 expression was more strongly related to PERK expression (P=0.000) than to IRE1 (P=0.044) or ATF6 expression (P=0.009) (Table 1) (Figures 4A-4D). The relationship between the levels of these two proteins in the same tissue and the clinicopathological parameters of the 72 HCC patients is presented in Table 2. Simultaneous expression of Beclin1 and PERK was not correlated with gender, age, history of hepatitis, history of cirrhosis, tumor size, or degree of differentiation but closely related to clinical stage (P = 0.044) (Table 2). Twenty-two patients were followed up for survival time, including 11 patients with simultaneous PERK and Beclin1 expression. The shortest overall survival time was 0.5 months, and the longest overall survival time was 34.5 months. In the matched PERK- and Beclin1-negative group, which included 11 cases, the shortest overall survival time was 4.25 months, and the longest overall survival time was 46 months. Kaplan-Meier survival analysis was used to generate survival curves for the PERK/Beclin-positive and PERK/Beclin-negative groups (Figure 4E); the median survival time in these two groups was 16.5 months and 27.5 months, respectively. The difference in overall survival time between the two groups was statistically significant (p=0.034). These data indicated the existence of a strong relationship between ER stress and autophagy; moreover, patients with both PERK and Beclin1 expression have more advanced disease and a shorter overall survival time.

### Sorafenib-induced ER stress is upstream of autophagy

**Figure 5:**
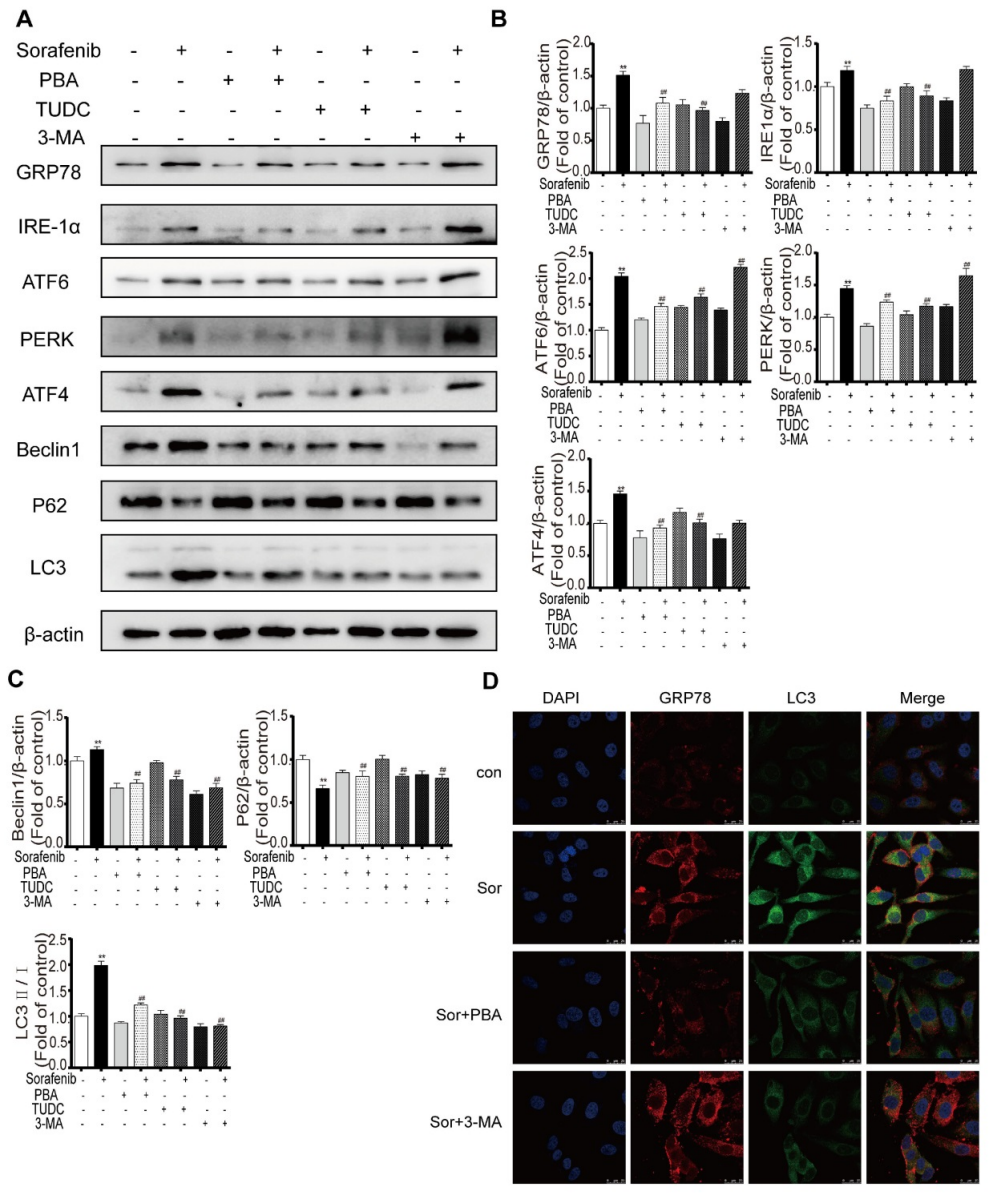
** Sorafenib-induced ER stress is upstream of autophagy.** (A) Representative Western blotting results of GRP78, IRE1α, ATF6, PERK, ATF4, LC3, P62 and Beclin1 when ER stress and autophagy were inhibited during sorafenib treatment. (B, C) The relative band density was measured by ImageJ. **P < 0.01 compared to control; ^##^P < 0.01 compared to sorafenib. (n = 3). (D) Immunofluorescence and colocalization of GRP78 with LC3 by confocal microscopy.

To explore the crosstalk between ER stress and autophagy in the background of sorafenib treatment, the representative ER stress inhibitors PBA and TUDC and the representative autophagy inhibitor 3-MA were used as important tools in our study. Western blot results showed that compared with sorafenib alone, sorafenib combined with PBA or TUDC restrained ER stress, as indicated by the decreased expression of GRP78, PERK, IRE1α, ATF6, and ATF4, and suppressed autophagy activity, as evidenced by the declines in the LC3-II/I ratio and in Beclin1. However, 3-MA alone suppressed autophagy without affecting ER stress (Figures 5A-5C). Consistently, the detection of protein expression abundance based on immunofluorescence intensity using laser confocal microscopy showed that PBA reduced the fluorescence intensity of both GRP78 and LC3B (Figure 5D). Together, the above results revealed that sorafenib-induced ER stress triggers autophagy.

### Sorafenib-induced ER stress triggers autophagy via the PERK pathway

**Figure 6:**
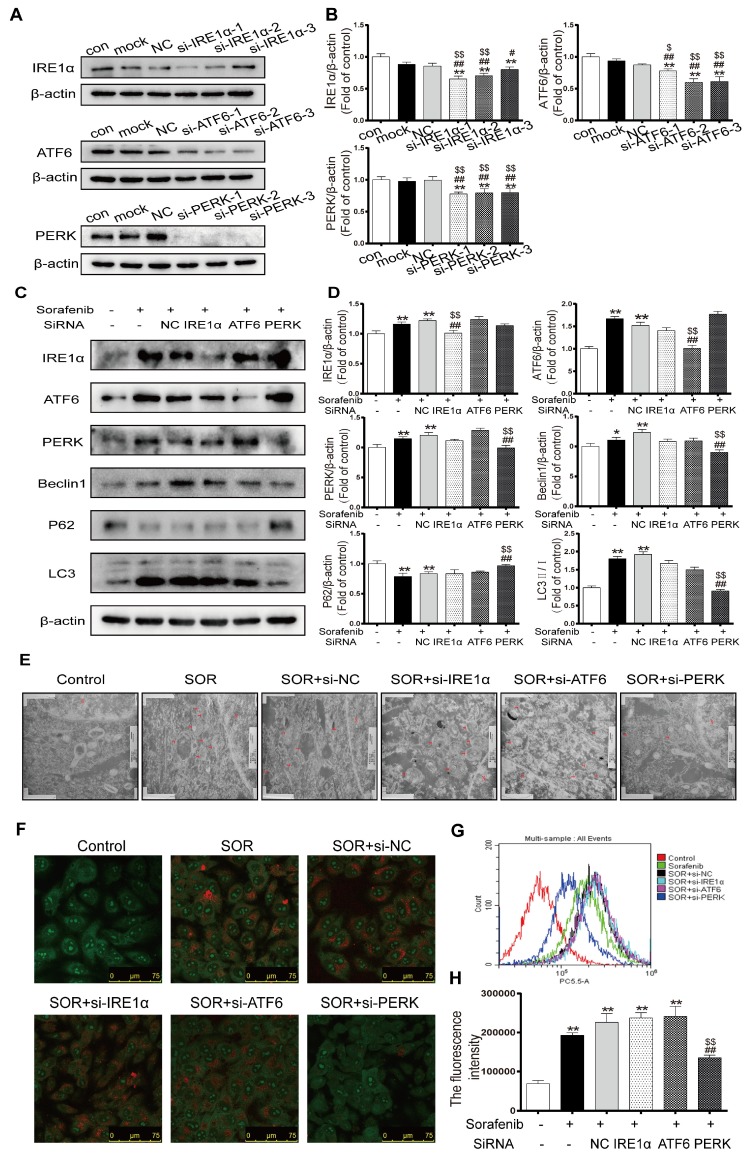
** Sorafenib-induced ER stress triggers autophagy through the PERK pathway.** (A, B) Western blot analysis of the most effective sequences to silence the UPR pathway. **P < 0.01 compared to control; ^##^P < 0.01, ^#^P < 0.05 compared to mock; ^$^P < 0.05, ^$$^P < 0.01 compared to NC. (n = 3). (C, D) Western blot analysis of IRE-1, ATF-6, PERK, P62, Beclin1 and LC3 expression after the three UPR pathways were knocked down in sorafenib-treated cells. (E) Autolysosomes were observed by transmission electron microscopy. (F) Acridine orange images were taken after the UPR pathway was knocked down in sorafenib-treated cells. (G) AVO quantification by flow cytometry analysis. (H) Mean orange fluorescence intensity. **P < 0.01 compared to control; ^##^P < 0.01 compared to sorafenib; ^$$^P < 0.01 compared to NC. (n = 3).

To further investigate the underlying mechanisms in ER stress by which the UPR pathway activates autophagy in the context of sorafenib treatment, HepG2 cells were transfected with specific siRNAs directed against all three UPR pathways, followed by observation of autophagy activity. First, we chose the most effective interference sequence among three candidates by Western blotting (Figures 6A-6B). Western blotting revealed downregulation of the protein expression levels of the autophagy markers LC3B-Ⅱ and Beclin1 and upregulation of P62 after exposing cells to PERK-siRNA and sorafenib (Figures 6C-6D). Conversely, knockdown of ATF6 or IRE1α did not suppress the sorafenib-induced upregulation of LC3 conversion. Furthermore, electron microscopy provided indisputable evidence that PERK knockdown obviously reduced autophagic vacuolar accumulation after 48 h of exposure to sorafenib (Figure 6E). Consistently, AVOs detected using acridine orange staining were reduced significantly in PERK siRNA-transfected HepG2 cells compared with other siRNA-transfected cells (Figure 6F). Quantification of AVOs by FACS analysis demonstrated that PERK knockdown significantly reduced the autophagic vesicle content after 48 h of sorafenib exposure, as indicated by the reduced mean orange fluorescence intensity (Figures 6G-6H).

### The PERK-ATF4-Beclin1 pathway plays an important role in ER stress-related autophagy

**Figure 7:**
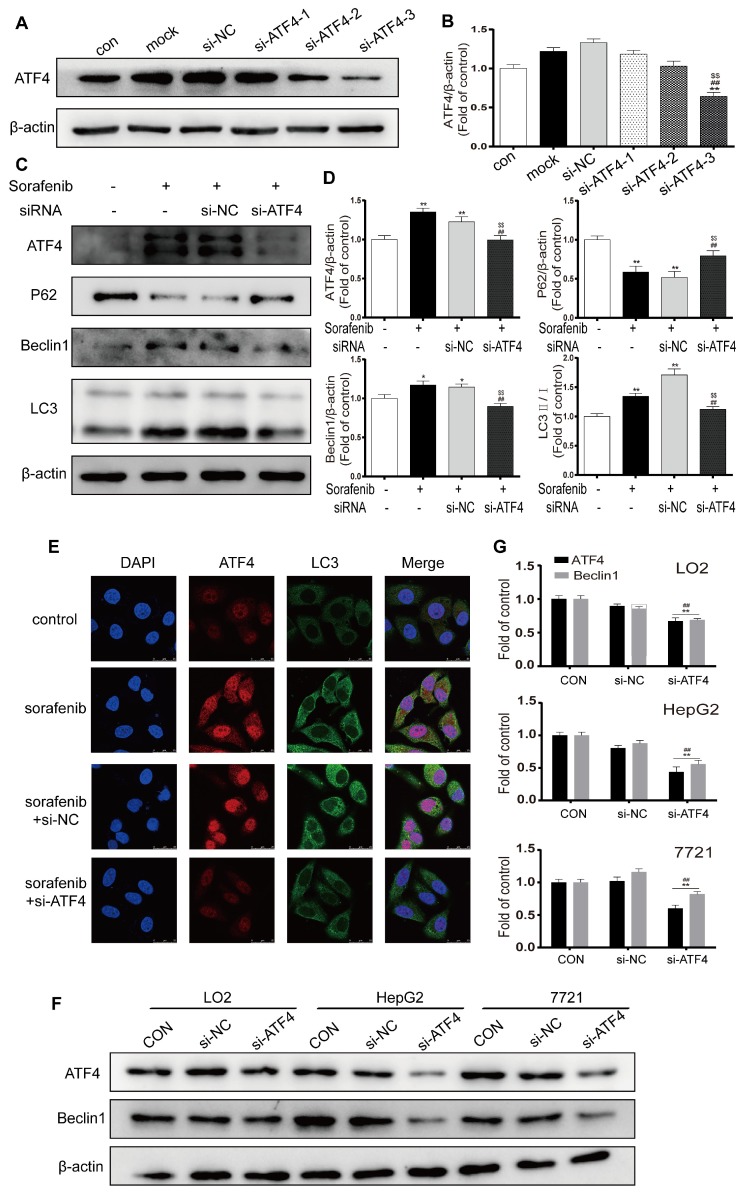
** The PERK-ATF4-Beclin1 pathway plays an important role in ER stress-related autophagy.** (A, B). Western blot analysis of the most effective sequences to silence ATF4. **P < 0.01 compared to control; ^##^P < 0.01 compared to mock; ^$$^P < 0.01 compared to NC. (n = 3). (C, D) Western blot analysis of ATF4, P62, Beclin1 and LC3 expression after ATF4 was knocked down in sorafenib-treated cells. *P < 0.05, **P < 0.01 compared to control; ^##^P < 0.01 compared to sorafenib; ^$$^P < 0.01 compared to NC. (n = 3). (E) The expression of ATF4 and LC3B was examined by immunofluorescence via confocal microscopy. (F, G) Western blot analysis of ATF4 and Beclin1 expression after ATF4 was knocked down in LO2, HepG2, and 7721 cells. **P < 0.01 compared to control; ^##^P < 0.01 compared to NC. (n = 3).

ATF4, a transcription factor that functions downstream of the PERK pathway, has been reported to modify the expression of multiple autophagy-related genes 28. We chose the most effective ATF4-siRNA sequences through Western blot analysis (Figures 7A-7B). After cells were exposed to ATF4-siRNA and sorafenib, Western blotting revealed that LC3B-Ⅱ and Beclin1 were downregulated and that P62 was upregulated (Figures 7C-7D). In the presence of sorafenib, the fluorescence intensity of both ATF4 and LC3B increased in HepG2 cells; after ATF4 was knocked down with siRNA, the fluorescence intensity of LC3B also decreased (Figure 7E). To examine the crosstalk between ATF4 and Beclin1, the human hepatocyte cell line LO2 and the HCC cell lines HepG2 and 7721 were transfected with the most effective ATF4-siRNAs, and Western blotting was conducted to detect Beclin1 expression. The results confirmed that Beclin1 was downregulated in the presence of ATF4 knockdown in LO2, HepG2, and 7721 cells (Figures 7F-7G). These findings indicate that sorafenib-induced ER stress triggers autophagy, possibly through the PERK-ATF4-Beclin1 pathway.

### Melatonin inhibits autophagy via the PERK-ATF4-Beclin1 pathway

**Figure 8:**
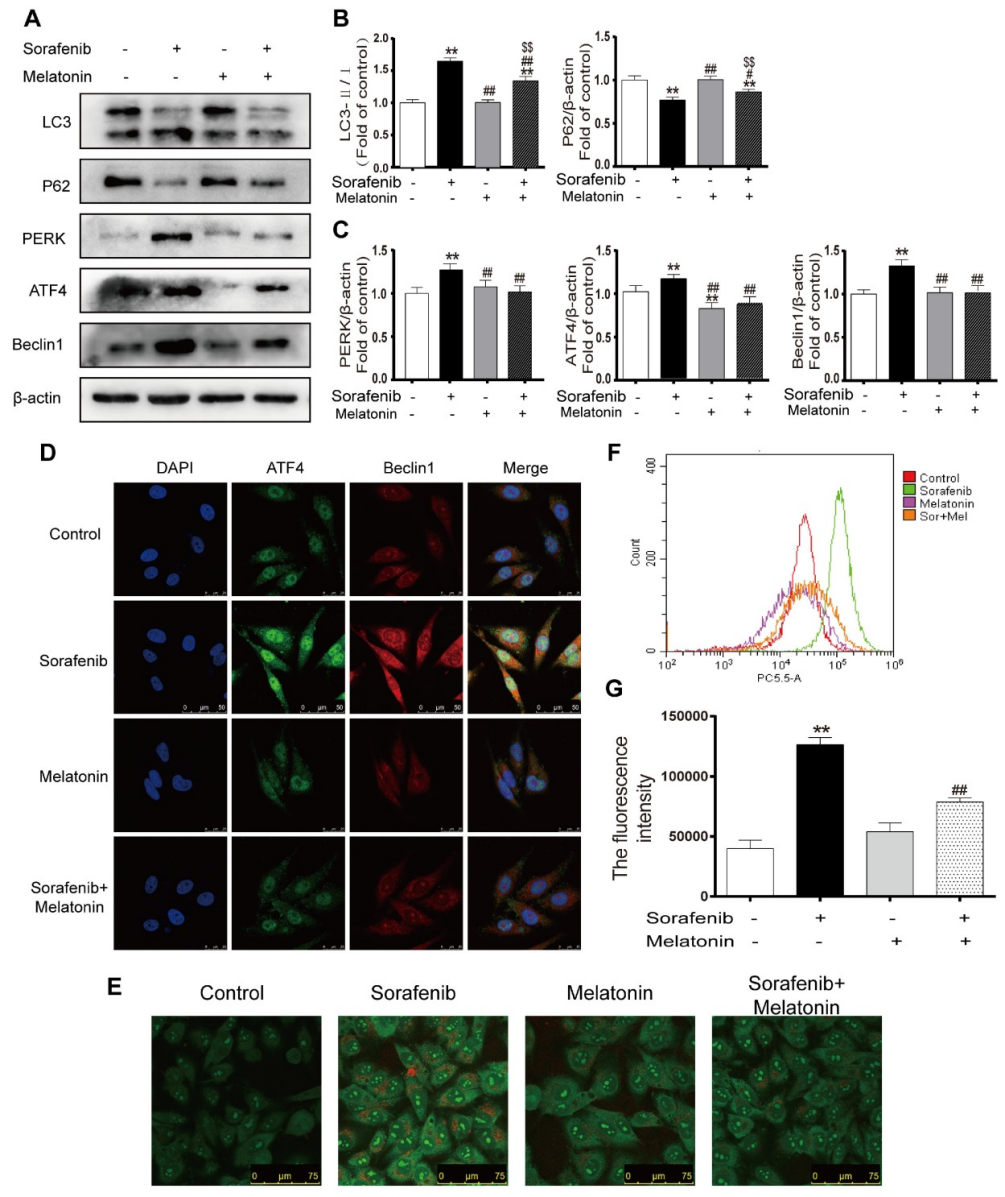
** Melatonin inhibits autophagy via the PERK-ATF4-Beclin1 pathway and increases the sensitivity of HCC cells to sorafenib.** (A, B, C) Western blot analysis of PERK, ATF4, P62, Beclin1 and LC3 expression in cells treated with sorafenib (10 μM) and/or melatonin (10^-5^ mol/L). (D) The expression of ATF4 and Beclin1 was examined by immunofluorescence via confocal microscopy. (E) Acridine orange images were taken after HepG2 cells were treated with sorafenib and/or melatonin. (F) AVO quantification by flow cytometry analysis. (G) Mean orange fluorescence intensity. **P < 0.01 compared to control; ^#^P < 0.05, ^##^P < 0.01 compared to sorafenib; ^$$^P < 0.01 compared to melatonin. (n = 3).

To elucidate whether melatonin increases sensitivity to sorafenib by inhibiting autophagy, the expression levels of LC3 and p62 were assessed in HepG2 cells by Western blotting. Notably, the combination of sorafenib and melatonin at 10^-5^ mol/L significantly reduced the LC3B-Ⅱ/Ⅰ ratio and enhanced P62 expression compared with sorafenib alone (Figures 8A-8B, Figures S1C-D). Furthermore, melatonin decreased the accumulation of AVO in the cytoplasm of sorafenib-treated HepG2 cells (Figure 8E). Flow cytometry analysis showed that the addition of melatonin decreased the red fluorescence intensity in sorafenib-treated cells (Figures 8F-8G). Accumulating evidence has revealed that melatonin can regulate the UPR pathway; thus, it is noteworthy that melatonin may potentially inhibit autophagy via the PERK-ATF4-Beclin1 pathway. Western blot analysis showed that melatonin downregulated the sorafenib-induced increases in PERK, ATF4, and Beclin1 expression (Figures 8A, 8C). Similar to the results of Western blot analysis, an immunofluorescence assay demonstrated that melatonin can decrease the intensity of fluorescein bound to Beclin1 and ATF4 in HepG2 cells exposed to sorafenib (Figure 8D). Considering the relationship between melatonin and PERK, ATF4, and Beclin1, it is reasonable to suggest that melatonin increases the sensitivity of HCC to sorafenib by inhibiting autophagy via the PERK-ATF4-Beclin1 pathway.

## Discussion

HCC is a malignant tumor with a high incidence in the digestive system. Globally, the incidence and mortality of HCC rank fifth and second among all cancers, respectively 29. Patients with early-stage HCC account for only a small fraction of the total morbidity; they are fortunate to be candidates for various treatments, such as surgical resection, transcatheter arterial chemoembolization (TACE) and liver transplantation 30, 31. However, most patients present with advanced, unresectable disease at diagnosis 32. Sorafenib, the only FDA-approved drug for the treatment of advanced HCC, inhibits tumor angiogenesis and cell proliferation by blocking the activity of multiple tyrosine kinases 33, 34. The effectiveness of continuous sorafenib application remains controversial, and acquired tolerance to sorafenib has been observed 35, 36. Among the mechanisms accounting for the ineffectiveness of sustained clinical cancer therapy, such as sorafenib therapy, apoptosis resistance is notable 26. The underlying mechanism of apoptosis resistance is complicated in HCC. Identification of the contributors to the development of apoptosis resistance during sorafenib treatment is a current research hotspot.

Several studies have shown that ER stress in tumor cells exposed to intrinsic and external factors can mediate apoptosis resistance through multiple signaling pathways and that this form of stress is closely related to chemotherapy resistance 37-39. Pathological conditions, including nutritional deficiencies, hypoxia and drug treatment, cause the aggregation and accumulation of unassembled and misassembled peptide chains in the ER lumen, a condition termed ER stress, and activate a range of stress-response signaling pathways referred to as the UPR 40. Our study showed that the expression levels of ER stress markers, including GRP78, PERK, IRE1α, ATF6, ATF4, XBP1S and CHOP, significantly increased with increasing concentration and duration of sorafenib treatment, suggesting that sorafenib can induce ER stress in liver cancer cells. In the present study, sorafenib combined with the ER stress inhibitors PBA and TUDC, which have been shown to act as chemical chaperones to decrease UPR signaling, meaningfully improved the percentage of liver cancer cells undergoing apoptosis compared with sorafenib alone. The above discoveries suggest that ER stress and the UPR induced by sorafenib are essentially cytoprotective responses to somewhat resist sorafenib-induced apoptosis.

The role of autophagy remains controversial in cancer treatment. It is now widely accepted that autophagy is a mechanism for escaping tumor cell death in which cells degrade misfolded and dysfunctional proteins and damaged organelles via the lysosomal pathway 41, 42. Our study revealed a link between apoptosis and autophagy under sorafenib treatment. We demonstrated that sorafenib induces autophagosome accumulation and causes the apoptosis of HCC cells. Furthermore, we observed that autophagy deficiency caused by 3-MA inhibition of autophagosome formation enhanced sorafenib-induced apoptosis. These findings suggest that autophagy responses potentially play a role in apoptotic resistance by clearing intracellular organelles damaged in response to sorafenib to restore normal intracellular homeostasis.

Research literature has demonstrated that activated ER stress can induce autophagy 9, 43. Additionally, the eIF2a-ATF4 pathway plays an indispensable role in autophagy-related gene transcription in response to stress 44. Autophagosome formation can be induced by ER stress through IRE1/JNK signaling 45. Our previous study revealed that XBP-1S, which is encapsulated by macrophage-derived exosomes, can transmit ER stress signals to HCC and induce autophagy. Consistent with a prior study, we observed that ER stress inhibitors (PBA and TUDC) significantly blocked sorafenib-induced autophagy, while specific autophagy inhibitors (3-MA) had no definitive effect on the activation of ER stress markers. These findings imply that ER stress is the starting point for autophagy, likely because autophagy activated by UPR clears and relieves the accumulation of unassembled and misassembled peptide chains caused by ER stress. Studies on sorafenib-induced ER stress in which the UPR pathway triggers autophagy are limited. An interesting discovery in the present study was that autophagy activity was markedly inhibited by silencing PERK in sorafenib-treated cells, while silencing ATF6 and IRE1α had no such effect.

ATF4 is a transcription factor produced in response to multiple microenvironmental stresses, including ER stress, and a component of the PERK pathway involved in the UPR 46, 47. Our results showed that autophagy activity was clearly reduced, while ATF4 and its upstream factor PERK were disrupted. Several studies have revealed that ATF4, as a transcription factor, is a master regulator that plays an important role by directly or indirectly eliciting the transcriptional activation of numerous autophagy-related genes, including LC3B, ATG5, ATG7, and Beclin1, in response to stress 44. Our experimental results extend the findings of these studies, indicating that Beclin1 expression is regulated by the PERK-dependent activation of ATF4. We identified the correlation between PERK and Beclin1 by evaluating tissue specimens from 72 patients with HCC. PERK and Beclin1 coexpression was significantly associated with more advanced clinical stage and shorter overall survival. Overall, our present study proposes a new mechanistic connection between ER stress and autophagy in which ER stress triggers autophagy via PERK-ATF4-Beclin1, which mediates a protective mechanism in HCC.

Our prior studies demonstrated that melatonin can overcome apoptosis resistance and sensitize HCC cells to ER stress-induced apoptosis, and we proposed that melatonin could enhance the sensitivity of HCC cells to sorafenib 48. We found that low concentrations of melatonin (10^-5^ mol/L) unexpectedly increased the antitumor effect of sorafenib but could not increase the apoptosis of HCC cells alone. This result can be readily explained, considering that some studies have reported that low concentrations of melatonin can inhibit autophagy and ER stress, and our team previously demonstrated that melatonin can increases the anti-tumor effects of sorafenib by inhibiting autophagy 49-50. Additionally, our current research showed that sorafenib-induced autophagy and ER stress are protective mechanisms for HCC cell survival, and we inferred that a low concentration of melatonin increases the sensitivity of HCC cells to sorafenib by inhibiting ER stress-related autophagy. In accordance with this assumption, we observed LC3-II degradation and increased p62 expression following treatment with sorafenib combined with low-dose melatonin compared with sorafenib alone. Next, we addressed the possibility that melatonin regulates ER stress-associated autophagy following sorafenib treatment via the PERK-ATF4-Beclin1 pathway. Surprisingly, our present results showed that combined treatment with melatonin and sorafenib caused the degradation of PERK, ATF4, and Beclin1 compared with treatment with sorafenib alone. Our present study suggests that melatonin inhibits autophagy via the PERK-ATF4-Beclin1 pathway to improve the sensitivity of liver cancer cells to sorafenib.

In conclusion, to our knowledge, this study is the first to show that melatonin regulates ER stress-induced autophagy via the PERK-ATF4-Beclin1 pathway to overcome apoptosis resistance and increase the sensitivity of HCC cells to sorafenib. Our findings provide new insight into the molecular mechanisms of apoptosis resistance in sorafenib treatment and suggest that melatonin may be a promising agent and that the PERK-ATF4-Beclin1 pathway may be a target for the treatment of HCC.

## Figures and Tables

**Table 1:** The expression of Beclin1 is more likely to be associated with the expression of PERK than with IRE-1 and ATF6 in hepatocellular carcinoma patients

Beclin1	PERK					IRE1					ATF6			
	+	-	χ^2^	*P-*value		+	-	χ^2^	*P-*value		+	-	χ^2^	*P-*value
Positive	35	4	-	0.000		31	8	4.044	0.044		34	5	6.732	0.009
Negative	3	30				19	14				20	13		

+: Positive; -: Negative; PERK: protein kinase RNA-like endoplasmic reticulum kinase; ATF-6: activating transcription factor 6; IRE1: inositol-requiring enzyme 1

**Table 2:** Association of clinicopathological features and Beclin1- and PERK- matched expression

Clinical pathologial factors	Total (N=72)	PERK and beclin1 matched		χ2	*P-*value
		Positive (N=35)	Negative (N=37)		
Gender, N(%)				0.891	0.345
M	53(73.6)	24(68.6)	29(78.4)		
F	19(26.4)	11(31.4)	8(21.6)		
Age, N(%)				0.607	0.436
≤60	44(61.1)	23(65.7)	21(56.8)		
>60	28(38.9)	12(34.3)	16(43.2)		
History of hepatitis ,N(%)				0.445	0.505
N	24(33.3)	13(37.1)	11(29.7)		
Y	48(66.7)	22(62.9)	26(70.3)		
History of cirrhosis, N(%)				0.459	0.498
N	42(58.3)	19(54.3)	23(62.2)		
Y	30(41.7)	16(45.7)	14(37.8)		
Tumor size, N(%)				1.946	0.378
<5cm	20(27.8)	10(28.6)	10(27.0)		
5-10cm	36(50.0)	15(42.9)	21(56.8)		
≥10cm	16(22.2)	10(28.6)	6(16.2)		
Clinical stages, N(%)				4.055	0.044*
Ⅰ/Ⅱ	53(73.6)	22(62.9)	31(83.8)		
Ⅲ/Ⅳ	19(26.4)	13(37.1)	6(16.2)		
Degree of differentiation, N(%)				2.053	0.358
high	22(30.6)	8(22.9)	14(37.8)		
moderate	34(47.2)	19(54.2)	15(40.6)		
poor	16(22.2)	8(22.9)	8(21.6)		

## Abbreviations

HCC: hepatocellular carcinoma
ER: endoplasmic reticulum
PERK: PKR-like ER stress kinase
ATF4: activating transcription factor 4
TKI: tyrosine kinase inhibitor
IRE1: inositol-requiring transmembrane kinase and endonuclease 1
ATF6: activating transcription factor 6
UPR: unfolded protein response
DMEM: Dulbecco's modified Eagle medium
PVDF: polyvinylidene difluoride
TUNEL: terminal deoxynucleotidyltransferase-mediated dUTP-biotin nick end labeling
3-MA: 3-methyladenine
PBA: phenylbutyrate
TUDC: tauroursodeoxycholate
SDS-PAGE: sodium dodecyl sulfate‑polyacrylamide gel electrophoresis
AVO: acidic vesicle organelle
TACE: transcatheter arterial chemoembolization
